# Next generation sequencing technologies for next generation plant breeding

**DOI:** 10.3389/fpls.2014.00367

**Published:** 2014-07-30

**Authors:** Soham Ray, Pratik Satya

**Affiliations:** ^1^Crop Improvement Division, Central Rice Research InstituteCuttack, India; ^2^Crop Improvement Division, Central Research Institute for Jute and Allied FibresKolkata, India

**Keywords:** next generation sequencing, plant breeding, single nucleotide polymorphism, genotyping, marker discovery

As a term, “next generation plant breeding” is increasingly becoming popular in crop breeding programmes, conferences, scientific fora and social media (Schnable, 2013). Being a frontier area of crop science and business, it is gaining considerable interest among scientific community and policymakers and funds flow from entrepreneurs and research funding agencies. Plant breeding is a continuous attempt to alter genetic architecture of crop plants for efficient utilization as food, fodder, fiber, fuel or other end uses. Although the scientific concepts in plant breeding originated about 100 years ago, domestication and selection of desirable plants from prehistoric periods have contributed tremendously to ensure human food security (Gepts, 2004). During the past few decades, well supported crop improvement programmes for major crops started reaping benefits from cutting edge technologies of biological sciences, particularly in the form of molecular markers and transgenic crop development, which in combination with conventional phenotype based selection, defines the current generation plant breeding practices. Different types of molecular markers have been developed and extensively used during the last three decades for identifying linkage between genes and markers, discovering quantitative trait loci (QTLs), pyramiding desired genes and performing marker assisted foreground and background selections for introgression of desired traits (Varshney and Tuberosa, 2007). However, these markers are based mostly on electrophoretic separation of DNA fragments, which limits detection of genetic polymorphism. In large plant breeding populations, genotyping may take up several months depending on marker system, adding more cost to genotyping. The next generation plant breeding would thus demand more efficient technologies to develop low cost, high-throughput genotyping for screening large populations within a smaller time frame.

With the availability of whole genome sequences (WGS), the perspective of identification of DNA markers has shifted from fragment based polymorphism identification to sequence based single nucleotide polymorphism (SNP) identification to expedite the marker identification process and to increase the number of informative markers. But the WGS technologies based on Sanger sequencing are time consuming, costly and provide information only on the target individual, which have limited its use in specific gene discovery. Its direct use in large breeding populations is limited by time and cost factors. The advent of next generation sequencing (NGS) technologies and powerful computational pipelines has reduced the cost of whole genome sequencing by many folds allowing discovery, sequencing and genotyping of thousands of markers in a single step (Stapley et al., 2010). NGS has emerged as a powerful tool to detect numerous DNA sequence polymorphism based markers within a short timeframe (Figure S1), growing as a powerful tool for next generation plant breeding.

The initial steps of NGS based marker development involve library construction prior to sequencing. Several targeted marker discovery techniques have been devised using NGS platforms which involve partial representation of the genome and those can be utilized even in absence of prior knowledge on WGS (Figure 1). Based on the approaches, partial genome representation libraries are either (i) complexity reduced representation libraries constructed by using restriction enzymes, or (ii) sequence capture libraries without involving restriction digestion. The first group includes reduced-representation libraries (Gore et al., 2009), complexity reduction of polymorphic sequences (Mammadov et al., 2010), restriction-site associated DNA sequencing (RAD-seq) (Pfender et al., 2011), sequence based polymorphic marker technology (Sahu et al., 2012), multiplexed shotgun genotyping (Andolfatto et al., 2011), and genotyping-by-sequencing (GBS) (Elshire et al., 2011). The second group includes technologies like molecular inversion probe (Porreca et al., 2007), solution hybrid selection (Gnirke et al., 2009) and microarray-based genomic selection (Albert et al., 2007). Sequence capture can also be performed for broad or specific targets in the genome such as exome sequencing (Teer and Mullikin, 2010) and sequencing of the genomic region associated with particular trait (Teer et al., 2010).

**Figure 1:**
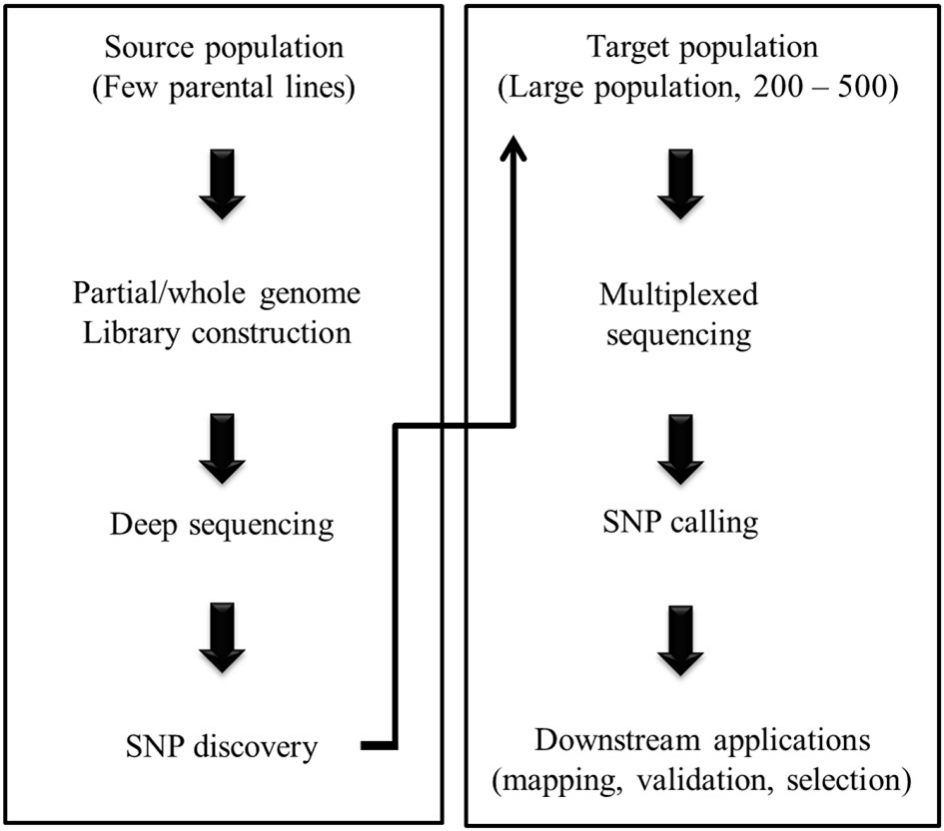
**A general outline of NGS assisted plant breeding**.

NGS technologies are already gaining widespread acceptability in the field of crop breeding. Many of the NGS based marker discovery techniques allow SNP discovery and genotyping simultaneously, speeding up the whole process (Figure 1). Furthermore, availability of gene and transcript sequence data at a large scale in the public domain allows development of genic molecular markers or functional markers. Of the various NGS technologies RAD-seq and GBS have already been proved to be effective for next generation plant breeding (Yang et al., 2012; Glaubitz et al., 2014). RAD-seq is basically a SNP based bulked segregants analysis technique where genomic DNA is sheared with a restriction enzyme of choice followed by ligation of barcoded adapter with molecular identifier (Pfender et al., 2011; Yang et al., 2012). Next, the processed DNA sample from multiple individuals (~20 individuals) are pooled and randomly sheared so that only a subset of generated fragments contain barcoded adapter. Another divergent adapter is ligated with the fragments for PCR. Divergent adapter ensures amplification of only those fragments containing both adapters. The resultant amplicons are sequenced using an Illumina platform. Finally, pooled samples with different identifiers are separated and SNPs are called using standard bioinformatic pipeline. This technique does not need *a priori* genome sequence information. RAD-seq tagged SNPs have been used to construct a linkage map in eggplant and to identify QTLs for anthocyanin pigmentation of the fruit (Barchi et al., 2012) and also to identify a resistance gene against anthracnose disease in lupin (Yang et al., 2012).

GBS has been used in development of high density map of 20000 SNPs in wheat and 34000 SNPs in barley (Poland et al., 2012a) and to map QTLs for spike architecture and reduced plant height in barley (Liu et al., 2014). It is a simple and highly multiplexed system which follows a modified RAD-seq based library preparation protocol for NGS that reduce sample handling, PCR and subsequent purification steps and completely excludes size fractionation of DNA using efficient barcoding technique. Unlike RAD-seq, the second adapter used in GBS is not a divergent one and hence it allows synthesis of amplicons flanked by any of the three adapter sequence combinations. Powerful bioinformatic pipelines have been established for GBS which can impute missing data utilizing available reference genome (Glaubitz et al., 2014). It allows simultaneous marker discovery and genotyping, and can be scaled up according to need.

If the reference genome sequence is available, the sequence based polymorphic marker technology is quite useful for marker discovery in targeted regions of a genome (Sahu et al., 2012). Short reads are mapped backed to the reference genome to identify putative SNPs. Assembly of multiple short reads assign confidence values to the identified SNPs. Once identified these SNPs are validated by wet lab experiments. The other technique which utilizes reference genome sequence is low coverage multiplexed shotgun genotyping where genomic DNA from multiple genotypes are pooled, sequenced and matched with reference genome with unique linked adapter. Pooling reduces sampling variation and increase efficiency of SNP identification.

The NGS technologies are pivotal to genomic selection, where performance of a target genotype can be predicted from its genomic estimated breeding value determined through statistical models derived using rigorous genotyping and phenotyping of a standard set of breeding population (Poland et al., 2012b). In addition to increasing selection efficiency in annual crop species, these methods are highly valuable for reducing duration of selection in perennial crops, where phenotypic expression of a trait may require several years. However, the complexity of plant breeding situations poses a great challenge to genomic selection, as the relationship between genotype and phenotype often depend on many macro- and micro-environmental factors. Accurate phenotyping and use of robust algorithm are thus of crucial importance to determine the genotype-phenotype relationship for application of genomic selection.

In spite of high potential, the achievements of NGS technologies have been limited to a few examples, most of which have been generated in by institutes with well-established genomic facilities. The technical expertise to extract usable information from huge sequence information presently is insufficient for large scale application of NGS technologies. The most important requirement for reaping benefits of NGS is to enable plant breeders to manage and extract information from huge genomic data. In addition, genomes with higher ploidy level, presence of homeologus sequences and more repetitive sequence poses problems for sequencing and assembly, but some of these problems may be addressed through upcoming technologies (Griffin et al., 2011; Teer et al., 2013). Successful construction of GBS map of wheat with 416,856 markers shows that the robust genetic map of polyploid crops can be constructed through NGS (Saintenac et al., 2013).

Cost of genotyping is another determining factor for adopting appropriate NGS technologies in plant breeding. Since crop breeding handles large population size, it is an expensive process itself. Choice between whole and partial genome sequencing would depend on the availability and judicious use of funds. The cost of WGS for a single genotype of three gigabase genome at 30X coverage is approximate $5000 (Hayden, 2014). Targeted sequencing approach like RAD-seq can sample 200000 SNPs in 100 individuals with same coverage depth at nearly 35-fold less cost compared to WGS of same 100 individuals (Davey et al., 2011). If the whole genome sequence is already available for the target organism the cost involved might further reduce by another 10–14 folds by using techniques like MSG or GBS. Presently, targeted sequencing seems to be more cost-effective option for large scale marker discovery, particularly in case of large and un-decoded genomes. The trend in sequencing technology development closely follows Moore's law (Wetterstrand, 2014), which indicates that the costs for WGS or NGS will reduce by several folds, and WGS may be preferred over partial genome sequencing in near future (Marroni et al., 2012). We expect that targeted sequencing approach would not be completely wiped out by the overwhelming flow of WGS; rather it would be a preferred choice for short term projects for strengthening next generation plant breeding. However, the additional associated cost for target enriched library preparation and bioinformatic analysis that precedes and succeeds the sequencing step, respectively, may not decrease as rapidly as the cost of sequencing the genome. The cost of data mining and efficiency to extract usable information may be more crucial than genotyping cost itself for application of NGS technologies in next generation plant breeding.

Apart from marker discovery, the NGS technologies are also being applied for targeted re-sequencing to identify domestication related genes by comparing the genome of crop species and their wild relatives (Henry, 2012), and also for genome wide selection studies to predict breeding value of traits, all of which have high potential to become application tools for the next generation plant breeders for development of superior cultivars. The ability to directly look into the genome sequences has revolutionized the science of plant breeding in the past few years, and NGS can serve as a worthy weapon for the next generation plant breeders to mitigate the rising demand of food, fiber and fodder in the coming decades. However, it may require some incubation period before this remarkable but complex technology can provide dividends to next generation plant breeders.

## Conflict of interest statement

The authors declare that the research was conducted in the absence of any commercial or financial relationships that could be construed as a potential conflict of interest.
