# Supplementary figures and images for: Next generation sequencing technologies for next generation plant breeding
Source: Front Plant Sci. 2014 Jul 30;5:367. doi: 10.3389/fpls.2014.00367 (PMC4115663; doi:10.3389/fpls.2014.00367)

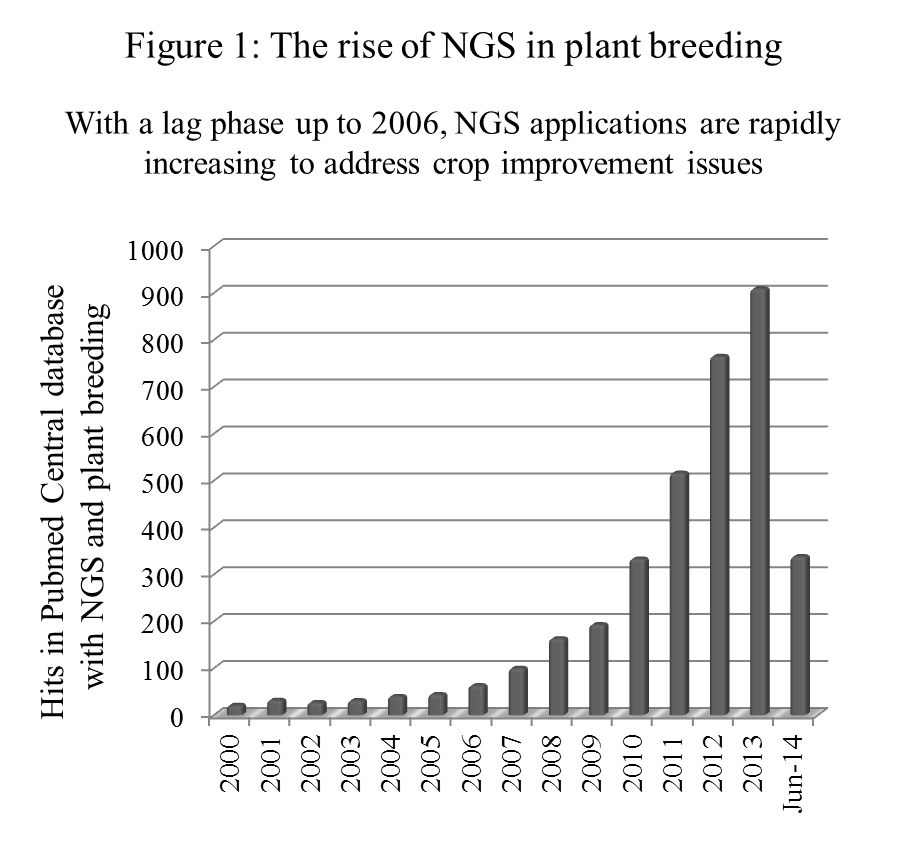

Supplement: Supplementary file 1 [file Image1.JPEG]
